# Neurological effects of hemodialysis on white matter microstructure in end-stage renal disease

**DOI:** 10.1016/j.nicl.2021.102743

**Published:** 2021-07-01

**Authors:** Junya Mu, Liang Ma, Shaohui Ma, Dun Ding, Peng Li, Xueying Ma, Ming Zhang, Jixin Liu

**Affiliations:** a Center for Brain Imaging, School of Life Science and Technology, Xidian University, Xi'an 710126, People's Republic of China; b Engineering Research Center of Molecular & Neuroimaging, Ministry of Education, Xi'an 710126, People's Republic of China; c Department of Medical Imaging, Second Affiliated Hospital of Xi’an Jiaotong University, Xi'an 710061, People's Republic of China; d The Affiliated Hospital of Inner Mongolia Medical University, Hohhot 010000, People's Republic of China; e Department of Medical Imaging, First Affiliated Hospital of Xi’an Jiaotong University, Xi'an 710061, People's Republic of China; f Department of Medical Imaging, Shaanxi Nuclear Geology 215 Hospital, Xianyang, People's Republic of China

**Keywords:** End-stage renal disease, Hemodialysis, White matter, Working memory, Neuroprotective effect

## Abstract

**Objectives:**

To detect the effects of hemodialysis (HD) on the central nervous system (CNS), the present study forces the memory storage capacity and the difference in white matter (WM) microstructure characteristics among end-stage renal disease (ESRD) participants before HD initiation (ESRD-BHD), ESRD participants with maintenance HD (ESRD-MHD), and healthy participants (HCs).

**Methods:**

Between 2016 and 2018, 56 ESRD-BHD, 39 ESRD-MHD, and 56 HCs were recruited for this study. The fractional anisotropy (FA) of tractography streamlines within the working memory network was investigated using a novel along-tracts analysis method. The relationship between WM microstructure and working memory scores, measured from an n-back task, were detected by multiple correlation analysis.

**Results:**

As compared with HCs, a significantly lower FA was found along part of the WM in the working memory network in ESRD-BHD. In the group-difference location of ESRD-BHD and HCs, the FA of ESRD-MHD was reversed to normal levels in HCs. However, the FA in a new location was differentially reduced across groups: highest in HCs, intermediate in ESRD-BHD, and lowest in ESRD-MHD. Correlation analysis showed that a longer reaction time correlated to a lower FA, according to the following pattern: ESRD-BHD > ESRD-MHD > HCs.

**Conclusion:**

Despite the persisting abnormal brain structure, our findings suggest HD has a neuroprotective effect in ESRD patients.

## Highlights

• Longer reaction time of n-back task was correlated to a lower diffusion property in ESRD patients.
• Diffusion property of HD population was reversal to normal levels in HCs.
• HD has a neuroprotective effect on CNS for ESRD patients.

## 1 Introduction

End-stage renal disease (ESRD) is defined by an eGFR < 15 ml/min/1.73 m^2^, and patients with ESRD require dialysis to support life (Lu et al., 2015). Although the prognosis of ESRD patients with dialysis remains poor, their increasing life expectancy has shifted medical attention from life-threatening emergencies to long-term complications and sequelae (such as cognitive decline due to brain structure abnormalities) (Viggiano et al., 2020a, Voskamp et al., 2019). The relationship between dialysis and the risk of cognitive function decline needs to be considered to address the considerable medical expenditure resulting from the lack of attention to brain structure dysfunction in ESRD patients (Lu et al., 2015). Despite the longstanding interest on the effects of dialysis on the brain, the effects of hemodialysis (HD) on the cognitive decline related to brain structure abnormalities is still largely unclear.

Emerging evidence has demonstrated a higher risk of central nervous system (CNS) dysfunction, blood–brain barrier disruption, and cerebrovascular disease in the dialysis population (Lu et al., 2015, Mu et al., 2018). However, studies also suggested that dialysis could remove metabolites from the blood, which may mitigate the side effects of uremic toxins in the CNS (Harhay et al., 2018, Li et al., 2018). Differences in study subjects and stage of kidney disease may be the major causes for these contradictory results. Most research on kidney disease related to CNS dysfunction focused on the comparison between long-term maintenance dialysis patients and healthy controls (Jager et al., 2008, Mark et al., 2018, Mu et al., 2018, Polinder-Bos et al., 2018). Therefore, they might report mixed effects on the CNS, overlooking the independent effect of dialysis and ESRD on the brain. This could be avoided by including baseline data from the point of dialysis initiation to long-term maintenance.

Working memory, as a higher-order cognitive function, is associated with mortality, hospitalization rates, and quality of life in ESRD patients (Yarkoni et al., 2011). To remain independent while receiving dialysis, patients must be able to accurately monitor their own activities (diet and medication), requiring basic working memory capacity. Working memory deficits are a distinguishing feature of preclinical stages even leading to Alzheimer’s disease (Kumar et al., 2017). The brain white matter (WM), which provides the wiring of the extensive neural networks activated during working memory tasks (Lazar, 2017), becomes vulnerable by blood–brain barrier disruption and uremic toxins in ESRD patients (Lu et al., 2015, Pierpaoli et al., 2001). Structural neurologic imaging studies showed reproducible findings of widespread and symmetrical WM microstructural changes in ESRD patients (Chou et al., 2013, Mu et al., 2018, Zhang et al., 2015). Hence, focus on the working memory, related WM will benefit to understand the abnormality in the CNS of ESRD patients.

Previously, we have shown that ESRD patients before dialysis initiation (ESRD-BHD) have an altered WM microstructure characteristic compared to healthy controls (HCs) (Mu et al., 2020). In the present study, we aimed to investigate the effect of HD on WM microstructure along-tracts within the working memory-related brain network. We compared the working memory-related streamline tractography between a cohort of prevalent ESRD-BHD and ESRD patients with long-term maintenance dialysis (ESRD-MHD). Multiple correlation analysis was used to examine the relation between the WM microstructure and the working memory scores which were measured from an n-back task.

## 2 Materials and Methods

All research procedures were approved by the Institutional Review Board of the First Affiliated Hospital of the Medical College at Xi'an Jiao tong University and conducted in accordance with the Declaration of Helsinki. All subjects provided written, informed consent after the experimental procedures had been fully explained.

### 2.1 Participants

Thirty-nine ESRD-MHD, 56 ESRD-BHD, and 56 HCs were recruited. Three groups were all matched in age, education, and gender. Eighty ESRD patients had kidney puncture biopsy. The underlying cause of ESRD was mesangiocapillary glomerulonephritis (32 patients), endocapillary proliferative glomerulonephritis (24 patients), sclerosing glomerulonephritis (4 patients), focal segmental glomeruloselerosis (12 patients), IgA nephropathy (4 patients), and membranous nephropathy (4 patients). Fifteen ESRD patients had a history of glomerulonephritis without kidney puncture biopsy, so we could not identify the glomerulonephritis type. All ESRD patients had a GFR < 15 ml/min/1.73 m^2^ and required renal replacement therapy; ESRD-MHD were undergoing HD for > 12 months; and ESRD-BHD were upcoming for their first HD. The exclusion criteria for all subjects were as follows: (a) macroscopic brain T2-visible lesions (regardless of size) on MRI scans, (b) existence of a neurological disease, (c) physical deformities, (d) alcohol, nicotine, or drug abuse, (e) diabetic nephropathy or primary hypertensive nephropathy, or (f) claustrophobia.

### 2.2 Laboratory examinations

Blood biochemistry tests including Creatinine, Urea, Cystatin C, Hemoglobin, Parathyroid Hormone, Calcium (Ca), Potassium (K), Sodium (Na), and Phosphorus (P) were conducted both in ESRD-MHD (before HD) and ESRD-BHD groups, three days before diffusion tensor imaging. No biochemistry tests were conducted in HCs.

### 2.3 Working memory evaluation by n-back task

Subjects viewed a continuous stream of sequential single random stimuli from a set of numbers. Each number was presented for 500 ms, with an inter-stimulus interval of 2,500 ms. Trials were divided into three conditions: 0, 1, and 2-back. In the 0-back condition, the target was a number “5.” The 1-back target was any number identical to that presented immediately before it in the series. In the 2-back condition, working memory load was increased and the target was any number identical to the number two back in the series. Breaks were allowed before blocks and each of the three n-back conditions was preceded by a practice session. For each condition, reaction time (RT) and accuracy (ACC) were recorded.

### 2.4 Data acquisition

All subjects were scanned on a 3.0 Tesla GE Excite scanner using an eight-channel coil (GE Medical Systems, Milwaukee, WI). DTI was obtained with a single-shot echo-planar imaging sequence. The diffusion sensitizing gradients were applied along two repeats of 30 non-collinear directions (b = 1,000 s/mm^2^) with five repeats of b0 (no diffusion weighted image). The imaging parameters were: 75 continuous axial slices with a slice thickness of 2 mm and no gap; field of view (FOV) = 256; TR = 9,400 ms; TE = 84 ms; and matrix size = 128 × 128, resulting in 2 mm isotropic voxels.

### 2.5 Data preprocessing

DTI data preprocessing was performed by using ExploreDTI (www.exploredti.com). Motion and EPI distortion correction, brain extraction, and fractional anisotropy (FA) were completed on ESRD-MHD, ESRD-BHD, and HCs data.

### 2.6 Working memory network

**Fig. 1:**
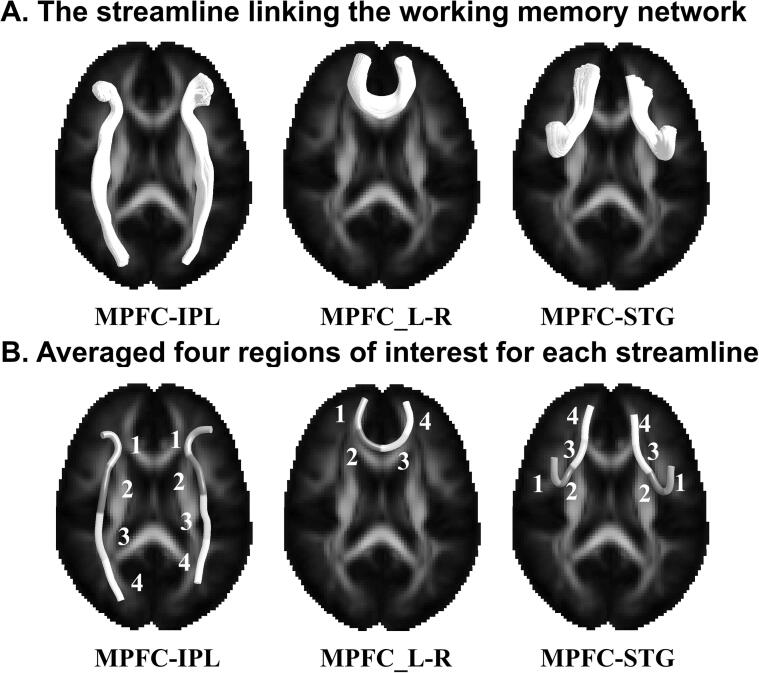
The streamlines linking the working memory network and four regions of interest were used for statistical analysis. (A). Five streamline trajectories connect the working memory network. (B). For statistical comparison, each streamline is divided into four regions of interest along consecutive tract sections. MPFC = medial prefrontal cortex; IPL = Inferior parietal lobule; STG = superior temporal gyrus.

In our study, the location of a working memory network was obtained from an online *meta*-analysis. Neurosynth is a platform for automatically synthesizing the results of various neuroimaging studies (https://www.neurosynth.org/). Working memory-related studies were initially selected by a search using the keywords “working memory.” Finally, a total of 1091 studies were selected for the analysis. The results were corrected for multiple comparisons using a cluster-based threshold z > 4.5 and *p* < 0.01 false discovery rate corrected. The results of this *meta*-analysis allowed us to identify regions consistently active in working memory across studies. We then focused on these brain regions to analyze the regional connections (Fig. 1A). The fiber bundle section process is explained in the “*Tractography atlas-based analysis (TABS)*” section.

### 2.7 TABS method

**Fig. 2:**
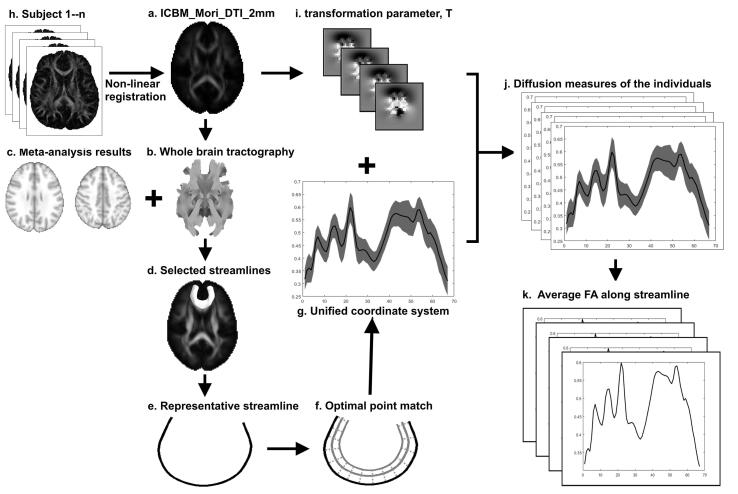
Framework of the tractography atlas-based analysis (TABS).

TABS is an along-tract statistics analysis method for detecting WM microstructural characteristics based on streamline tractography, as described in our previous study (Liu et al., 2017, Liu et al., 2019, Mu et al., 2018). This method facilitates an easy interpretation of results by directly reporting the resulting statistics on the streamlines. Specific steps are as follows (Fig. 2):

Step 1: To ensure the statistical analysis across subjects was in a common space, we leveraged the ICBM_Mori_DTI_2mm diffusion tensor template (Fig. 2A). Whole-brain tractography (Fig. 2B) was performed within the template with a step size of 1 mm, an angle threshold of 30, and a minimum tract length of 50 mm.
Step 2: The streamlines linking the regions in the *meta*-analysis results were selected from the tractography data. The streamlines from the bilateral medial prefrontal cortex (MPFC) to the superior temporal gyrus (STG), bilateral MPFC to the inferior parietal lobule (IPL), and left to right hemispheres of the MPFC were selected (Fig. 2C–D). Specifically, the fiber clusters were chosen from the whole-brain tractography maps of the ICBM_Mori_DTI_2mm template using manually defined inclusion, AND, OR, and exclusion with the ExploreDTI toolbox (www.exploredti.com) (Leemans et al., 2009).
Step 3: For each fiber bundle, a representative streamline was employed from the selected streamlines with the greatest local density weighted length (Fig. 2E). The representative streamline was then modeled by using the continuous arc length function.
Step 4: To ensure all streamlines can be represented by a unified coordinate system (Fig. 2G), the coordinates in the representative streamline were transformed to all other streamlines by using the optimal point match method (Fig. 2F).
Step 5: Non-linear transformation was then used to map the FA images of all subjects (Fig. 2H) to the template FA image to obtain the transformation parameter, T, from native space to template space (Fig. 2I). The unified coordinate system calculated in step 4 was warped back into the individual’s native space to collect the diffusion measures with T. The individual diffusion measures (Fig. 2J) were then averaged along the streamline (Fig. 2K).

### 2.8 Statistical analysis

Group differences in the subjects’ demographic information (age, years of education, emotional status) were analyzed using one-way ANOVAs, and post-hoc comparisons using *t*-tests. Sex differences were evaluated by using the χ2 test. Statistical threshold was set at *p* < 0.05.

Two-sample *t*-tests were used to investigate group differences of laboratory examinations between ESRD-MHD and ESRD-BHD. Statistical threshold was set at *p* < 0.05

For TABS, numerous streamlines from each fiber bundle were first averaged into a single streamline. Individual streamlines were then averaged over four regions of interest (ROI) (Fig. 1B). Permutation-based nonparametric inferences were used (5,000 random permutations) to calculate the between-group FA differences along the streamline among the ESRD-MHD, ESRD-BHD, and HCs. The threshold for statistical significance was *p* < 0.05 using threshold free cluster enhancement with the family wise error correction for multiple comparison corrections.

### 2.9 Correlation analysis between WM microstructure and working memory scores

“Behavior PLSC” was used to detect the correlation between WM of ESRD-MHD/ESRD-BHD/HCs and working memory scores (Krishnan et al., 2011). ESRD-MHD/ESRD-BHD/HC FA values were stored in a matrix denoted as X, with rows representing participants and FA value and columns representing spatial locations (i.e., voxels) within a WM. The working memory scores were represented by a matrix Y, in which rows represented ESRD-MHD/ESRD-BHD/HCs individuals and columns represented n-back RT. The clinical measures were repeated to obtain the same number of rows as in the DTI data block. Using “behavior PLSC,” a cross-correlation matrix (R) was computed by estimating the covariance between X and Y. Singular value decomposition of R was then performed, and by projecting the resulting left and right singular vectors onto the X or Y matrices, three pairs of latent variables (LVs) (DTI parameter × three working memory scores) with their corresponding singular values and voxel saliences were computed.

LV significance was assessed using permutation tests. A new data set (permutation sample) was obtained by randomly reordering the X rows but without changing Y/design matrix. The permutation sample was then recomputed in the PLSC model thereby obtaining new singular values. This procedure was repeated 5000 times and the likelihood of the singular values obtained in the original analysis being due to random chance determined.

## 3 Results

### 3.1 Demographic characteristics of subjects and working memory scores among ESRD-MHD, ESRD-BHD, and HCs

**Fig. 3:**
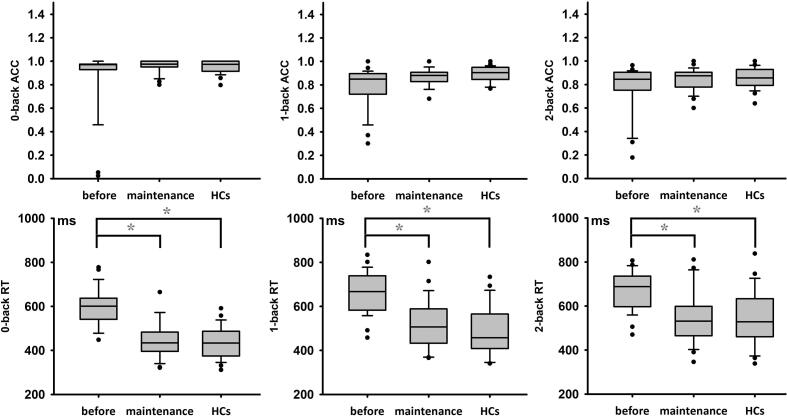
Box plot showing the working memory score in ESRD-MHD, ESRD-BHD, and HCs. Before = ESRD-BHD = ESRD patients before the initiation of HD; maintenance = ESRD-MHD = ESRD participants with maintenance HD; ESRD = End-stage renal disease; HD = hemodialysis; RT = reaction time; ACC = accuracy.

Demographics for ESRD-MHD, ESRD-BHD, and HCs are summarized in Table 1. There were no significant differences in age and years of education among three groups. Working memory scores among ESRD-MHD, ESRD-BHD, and HCs are summarized in Fig. 3. There were no significant differences in 0-back/1-back/2-back accuracy among ESRD-MHD, ESRD-BHD, and HCs. For the RT n-back, all conditions (1, 2, 3) follow the pattern ESRD-BHD > ESRD-MHD > HCs. Additionally, hemoglobin, urea, Na+, K+, Ca+, and parathyroid hormone showed significant differences between ESRD-MHD and ESRD-BHD (p < 0.05), but no difference was found for Creatinine, Cystatin C and P+ (p > 0.05) (Table 1).

**Table 1:** Demographic and clinical characteristics of HC, ESRD-BHD, and ESRD-MHD patients.

Variables	HCs N = 56	ESRD-BHD N = 56	ESRD-MHD N = 39	P-value	P1	P2	P3
***Demographic***							
Age(years)	38.15(1.79)	36.76 (2.48)	34.3(2.05)	0.231^a^	0.397^c^	0.084^c^	0.367^c^
Gender(M:F)	30 : 26	32:24	22:17	0.784^b^	–	–	–
Dialysis duration	–	–	30(4.62)	***–***	–	–	–
Education(years)	11.30(0.77)	12.51(1.25)	11.4 (0.91)	0.388^a^	0.642^c^	0.913^c^	0.679^c^
***Laboratory examinations***							
Hemoglobin(g/L)	–	87.18(2.72)	102.44(4.11)	–	–	–	0.002^c^^,^*
Creatinine(umol/l)	–	839.27(45.75)	939.76(50.96)	–	–	–	0.167^c^
Urea(μmol/L)	–	30.38(1.50)	23.48(1.69)	–	–	–	0.005^c^^,^*
Cystatin C(mg/L)	–	4.36(0.15)	4.59(0.26)	–	–	–	0.418^c^
Na+(mmol/L)	–	140.65(0.54)	144.48(0.63)	–	–	–	<0.001^c^^,^*
K+ (mmol/L)	–	4.56(0.11)	4.98(0.15)	–	–	–	0.026^c^^,^*
CA+(mmol/L)	–	1.96(0.03)	2.13(0.04)	–	–	–	0.007^c^^,^*
P+(mmol/L)	–	1.82(0.81)	1.78(0.08)	–	–	–	0.752^c^
Parathyroid hormone (pg/mL)	–	301.27(27.89)	747.72(93.68)	–	–	–	<0.001^c^^,^*

P1 = HCs vs. ESRD-BHD *P2* = HCs vs. ESRD-MHD *P3* = ESRD-BHD vs. ESRD-MHD.

Abbreviation: ESRD = end-stage renal disease; HCs = healthy controls; HD = hemodialysis; ESRD-BHD = ESRD before the initiation of HD; ESRD-MHD = ESRD participants with maintenance HD.

a The *P* values were obtained by using one-way analysis of variance.

b The *P* values were obtained by using the χ2 test.

c The *P* values were obtained by using a two-sample *t* test.

* indicate p < 0.05.

### 3.2 Along-tract statistical analysis

**Fig. 4:**
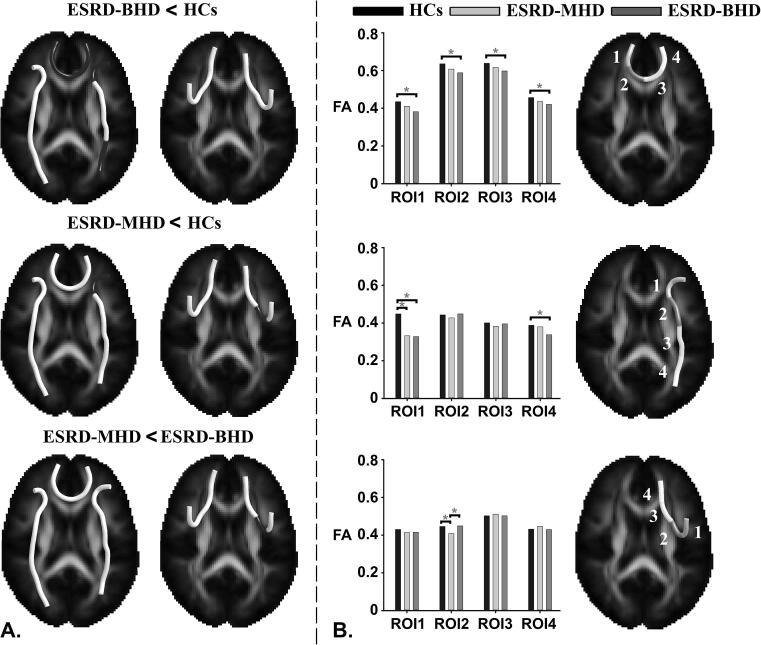
Significant FA differences for the working memory network in ESRD-BHD and ESRD-MHD relative to HCs. (A). Location of significant FA difference in ESRD-BHD and ESRD-MHD relative to HCs. (B). FA value in the four regions of interest along consecutive tract sections. ESRD-BHD = ESRD participants before the initiation of HD; ESRD-MHD = ESRD participants with maintenance HD; ESRD = End-stage renal disease; HD = hemodialysis. HC = healthy controls. *P-value < 0.05.

The difference in four average streamlines in ESRD-BHD and ESRD-MHD relative to HCs are shown in Fig. 4A, with corresponding histograms for each ROI (Fig. 4B). The statistical analysis for FA revealed a significant difference in MPFC-IPL-R and MPFC_L-R across the three groups: FA was highest in HCs, intermediate in ESRD-MHD, and lowest in ESRD-BHD. The FA of the ESRD-MHD tended to return to normal HC levels. However, in the MPFC-STG-R the FA order was different: FA was highest in HCs, intermediate in ESRD-BHD, and lowest in ESRD-MHD.

### 3.3 Associations between FA and working memory scores

**Fig. 5:**
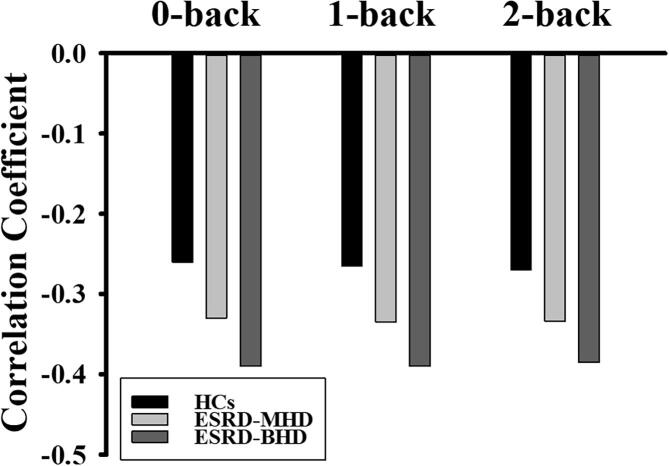
Multivariate analysis associating white matter to working memory score in ESRD-BHD, ESRD-MHD and HCs subjects, and showing the associations between FA and working memory reaction time found by a latent variable. The pattern shows far worse working memory (longer reaction time) correlates with lower FA. ESRD-BHD = ESRD participants before the initiation of HD; ESRD-MHD = ESRD participants with maintenance HD; ESRD = End-stage renal disease; HD = hemodialysis. HC = healthy controls; FA = fractional anisotropy.

A multivariate PLSC analysis showed that longer working memory RT correlated to lower FA. Additionally, a differential reduction in the correlation strength between FA and working memory RT was found (HC < ESRD-MHD < ESRD-BHD) (Fig. 5).

## 4 Discussion

The present study aimed to investigate the association of HD and working memory-related WM microstructure among ESRD-BHD, ESRD-MHD, and HCs. Comparing the three groups, the lowest FA in streamlines linking the working memory network were found in ESRD-BHD, with the reduction order of ESRD-BHD < ESRD-MHD < HCs. The PLSC correlation pattern between FA and working memory score showed a differential reduction in correlation strength (ESRD-BHD > ESRD-MHD > HCs). These results revealed intermediate FA levels in ESRD-MHD, suggesting that ESRD-MHD likely returns to the normal levels of HCs. Our study suggested that HD in ESRD-MHD may mitigate the side effects of uremic toxins on the working memory network. However, an additional difference with significantly lower FA relative to HCs was also found in ESRD-MHD, which may indicate a neuronal compensatory mechanism in ESRD-MHD, or a deteriorating effect of HD on the CNS. However, this finding needs to be further clarified.

### 4.1 Neuroprotective effects of HD on the working memory network

In our study, as compared with patients with ESRD before dialysis initiation, ESRD patients who underwent HD showed an improvement in working memory capacity. In clinical practice, HD is commonly initiated at some point after stage 5 CKD but before renal function collapses. The improvement in cognitive function after initiation of dialysis has been proved in several studies (Harhay et al., 2018, Li et al., 2018, Neumann et al., 2018). In some cross-sectional studies, researchers explored ESRD’s clinical assessment and found that memory performance improved after a single dialysis treatment (Harhay et al., 2018, Li et al., 2018). In a multicenter prospective cohort study, a dialysis population under a 2-year follow-up showed immediate memory improvements (Zhang et al., 2018), and an interventional study reported that patients on regular HD therapy experienced significant improvements in executive function and immediate memory after 12 months (Kurella Tamura et al., 2013). Our study is consistent with these studies and indicated that HD therapy improved working memory. Based on the kidney–brain crosstalk mechanism, kidney disease affects both the kidneys and the brain by shared vasoregulatory systems and humoral pathways. The brain and the kidney might interact through water channel dysregulation and accumulation of uremic toxins (Lu et al., 2015). HD replaces crucial kidney functions, such as filtration, secretion, and clearance of medications, all of which may relieve the negative effects of kidney disease-related risk features on the CNS (Neumann et al., 2018). Besides, previous studies found that these structural changes were associated with changes in blood laboratory examinations and that cognitive function improved after renal transplantation (Zhang et al., 2016). The recovery of renal function may underlie the cognitive improvement; hence, we inferred that HD replaces crucial kidney functions, which may have a neuroprotective effect on WM microstructure within the working memory network.

Regarding the impact of HD on the brain, there are also opposing findings. Earlier studies found worse cognitive function over time in dialysis patients. Some researchers focused on the relationship between cerebral blood flow and cognitive function, and reported that HD-related intradialytic cerebral hypoperfusion contributes to cognitive decline (Drew et al., 2019, Wolfgram, 2019). Consistent with the general population, the degree of WM damage in patients on HD also correlates with worse cognitive test scores, suggesting that cerebrovascular disease contributes to cognitive dysfunction in the HD population (Lu et al., 2015). One hypothesis suggests that HD removes urea more slowly from the brain than from plasma, creating an osmotic gradient resulting in cerebral edema (Silver et al., 1996); other hypotheses suggest that hemodynamic instability and cerebral ischemia are the reason for the cognitive impairment in the HD population (Lu et al., 2015). All these studies and hypotheses are inconsistent with our findings. Importantly, both the baseline and control groups in our study are different from previous research. In our study, the baseline consists of ESRD patients who did not undergo HD, and the control group includes ESRD patients with maintenance HD. According to a recent review paper, the mechanisms of cognitive dysfunction in CKD may be connected to the accumulation of uremic neurotoxins (Viggiano et al., 2020b). Based on our findings and previous studies, we hypothesized that CKD patients who experience reduced renal function and accumulation of uremia toxins may have progressive disruption of the cortical network and cognitive decline. An earlier study evaluated cognitive function among stage 3–5 CKD patients on HD and noted a graded decline in function with CKD progression (Kurella et al., 2004). The cognitive decline prevalence increases as a function of CKD stage and the cognitive dysfunction trend in patients with CKD may reach its most serious condition at end stages (Viggiano et al., 2020b). Based on early studies and the kidney–brain crosstalk mechanism, we assumed that the CNS dysfunction may be worst before dialysis initiation in ESRD patients (Lu et al., 2015). With the removal of uremia toxins, HD populations tend to restore their working memory capacity to general population level, but still lower than the non-CKD population. However, we did not compare the change of working memory over a shorter or longer HD duration. We cannot tell whether the course of working memory might reach a plateau or even decline at some point over a shorter or longer term. A reason for the inconsistent results may be the difference of stages in the CKD population between earlier studies and ours. But we consider it unlikely that the HD is the only having a negative effect on the CNS.

### 4.2 Neuronal compensatory mechanism or a deteriorating effect of HD on the CNS?

In our results, we also found a different FA order in MPFC-STG-R: FA was highest in HCs, intermediate in ESRD-BHD, and lowest in ESRD-MHD. The potential explanations of this finding are the following. First, the initiated maintenance dialysis may attenuate the encephalopathy caused by severe kidney failure, but this improvement may be outweighed by the processes contributing to cognitive decline, such as sudden hemodynamic shifts (Kurella Tamura et al., 2017). At what point working memory may reach a stable level, and when cognitive decline may take place while being treated with HD needs to be investigated in the future. The second possible explanation is based on a compensatory hypothesis, which suggests that the brain compensates in multiple ways for neural damage in an effort to function optimally (Han et al., 2007). Abnormal WM microstructural characteristics in the working memory network will limit working memory capacity. A PET study showed decreased metabolic activity in the MPFC of ESRD patients compared with healthy individuals (Song et al., 2008). Similarly, patients on HD have an increased number of connections to the MPFC (Qiu et al., 2014). These results seem to indicate a compensatory mechanism may have occurred in ESRD-MHD. However, further studies should be powered to evaluate how cognitive impairment might evolve over time on dialysis, and the specific adverse outcomes associated with different cognitive impairment domains. In addition, the altered microstructural properties may be due to the regional gray matter atrophy in ESRD patients. Some studies have reported that ESRD patients with maintenance HD show a reduced gray matter volume (Ding et al., 2018, Schaier et al., 2019). Future studies should investigate the relationship between streamline connections and gray matter alterations.

## 5 Limitations

Our study has several limitations which need to be addressed in future studies. First, we only focused on WM in the working memory network, finding that HD had an improvement effect on working memory capacity. Other measures of cognition should also be considered in the ESRD population. Further research needs to determine whether the neuroprotective effect of HD only implicates the working memory network or is also found in other regions. Second, the streamlines linking the working memory network were all whole regions of the MPFC, STG, and IPL. For the statistical analysis across subjects, numerous streamlines of each fiber bundle were averaged into a single streamline. The sub-streamlines connecting sub-regions may reveal the effect of HD on the CNS. Third, we only used FA to model fiber tract properties. The reason for not using MD, RD, and AD are that various measures difficult the interpretation of results and decrease statistical power and sensitivity. All these diffusion measures are interrelated with each other, and may share overlapping information. It is likely that the complex pathological phenomena altering the WM microstructure would simultaneously affect several diffusion parameters. Hence, it might be desirable to look at the combination analysis of all diffusion measures rather than each measure separately (Alnæs et al., 2018, Chamberland et al., 2019). Future studies should consider using the data fusion method (PCA, ICA) to comprehensively assess WM microstructure.

## 6 Conclusion

In summary, we found that in a large, diverse cohort of patients with ESRD with maintenance HD and before HD initiation, working memory decline was associated with altered WM microstructure of the working memory network. Our results suggest that HD has a neuroprotective effect on CNS dysfunction in ESRD patients. Additional studies are needed to determine how CNS dysfunction might evolve over time on dialysis.

## CRediT authorship contribution statement

**Junya Mu:** Writing - original draft, Conceptualization, Methodology, Software, Funding acquisition. **Liang Ma:** Formal analysis, Methodology, Software, Writing - review & editing. **Shaohui Ma:** Writing - review & editing, Investigation, Resources, Data curation. **Dun Ding:** Resources, Data curation. **Peng Li:** Resources, Data curation. **Xueying Ma:** Resources. **Ming Zhang:** Funding acquisition, Project administration. **Jixin Liu:** Funding acquisition, Writing - review & editing, Project administration.

## Declaration of Competing Interest

The authors declare that they have no known competing financial interests or personal relationships that could have appeared to influence the work reported in this paper.
